# Integrative analysis of transcriptome-wide association study and gene expression profiling identifies candidate genes associated with stroke

**DOI:** 10.7717/peerj.7435

**Published:** 2019-07-29

**Authors:** Jian Yang, Bin Yan, Yajuan Fan, Lihong Yang, Binbin Zhao, Xiaoyan He, Qingyan Ma, Wei Wang, Ling Bai, Feng Zhang, Xiancang Ma

**Affiliations:** 1 Clinical Research Center, The First Affiliated Hospital of Xi’an Jiaotong University, Xi’an, China; 2 Center for Brain Science, The First Affiliated Hospital of Xi’an Jiaotong University, Xi’an, China; 3 Department of Psychiatry, The First Affiliated Hospital of Xi’an Jiaotong University, Xi’an, China; 4 Health Science Center, Xi’an Jiaotong University, Xi’an, China

**Keywords:** Stroke, Gene ontology, Pathogenesis, Transcriptome-wide association study

## Abstract

**Background:**

Stroke is a major public health burden worldwide. Although genetic variation is known to play a role in the pathogenesis of stroke, the specific pathogenic mechanisms are still unclear. Transcriptome-wide association studies (TWAS) is a powerful approach to prioritize candidate risk genes underlying complex traits. However, this approach has not been applied in stroke.

**Methods:**

We conducted an integrative analysis of TWAS using data from the MEGASTROKE Consortium and gene expression profiling to identify candidate genes for the pathogenesis of stroke. Gene ontology (GO) enrichment analysis was also conducted to detect functional gene sets.

**Results:**

The TWAS identified 515 transcriptome-wide significant tissue-specific genes, among which *SLC25A44* (*P* = 5.46E−10) and *LRCH1* (*P* = 1.54E−6) were significant by Bonferroni test for stroke. After validation with gene expression profiling, 19 unique genes were recognized. GO enrichment analysis identified eight significant GO functional gene sets, including regulation of cell shape (*P* = 0.0059), face morphogenesis (*P* = 0.0247), and positive regulation of ATPase activity (*P* = 0.0256).

**Conclusions:**

Our study identified multiple stroke-associated genes and gene sets, and this analysis provided novel insights into the genetic mechanisms underlying stroke.

## Introduction

Stroke is the second leading cause of death and the third leading cause of disability-adjusted life-years lost worldwide (Feigin, Norrving & Mensah, 2017; Hankey, 2017). Causal risk factors, such as hypertension, hyperlipidemia, carotid stenosis, and atrial fibrillation, account for a substantial proportion of stroke risk (Bath & Krishnan, 2014; Bellolio, Gilmore & Ganti, 2014; Benjamin et al., 2018; ENOS Trial Investigators, 2015). However, the pathogenesis of stroke is still elusive. Family studies have indicated that genetic factors contribute substantially to the cause of stroke (Jerrard-Dunne et al., 2003). Recently, genome-wide association studies (GWASs) have identified multiple genetic variants associated with stroke (Gudbjartsson et al., 2009; Holliday et al., 2012; International Stroke Genetics Consortium et al., 2012; Kilarski et al., 2014;  SiGN & ISGC, 2016). Furthermore, the MEGASTROKE Consortium conducted a large-scale GWAS meta-analysis for stroke with almost all available samples; this analysis identified a number of relevant genetic loci (Malik et al., 2018). Although GWASs have yielded many causal genetic variants, the specific mechanisms underlying this disease have still not been identified.

Transcriptome-wide association studies (TWASs) have been recently proposed as a powerful approach to prioritize candidate risk genes underlying complex traits. Briefly, this approach can be viewed as a tool for predicting trait-associated gene expression based on GWAS summary data (Gusev et al., 2016). A set of individuals for whom both gene expression and genetic variation (single nucleotide polymorphisms (SNPs)) have been measured was used to calculate the cis-genetic component of expression weights. In recent years, TWASs have been widely applied to complex diseases, such as schizophrenia, Parkinson’s disease, and prostate cancer (Gusev et al., 2018; Mancuso et al., 2018; Ratnapriya et al., 2019). Studies have shown that this method is a powerful approach to predict candidate genes by integrating gene expression panels and GWAS summary data. However, much remained to be done to support these results.

Gene expression profiling is an approach to determine the pattern of genes expressed at the level of transcription. DNA microarrays or sequencing technologies have been applied to identify active genes for diseases. Typically, this approach provides experimental information for potential genes and may be a good complement to TWAS analysis. Moreover, the Gene Expression Omnibus (GEO) database has incorporated gene expression and hybridization array data for various diseases, providing a convenient basis for the integrative analysis of TWASs and gene expression profiling data.

Accordingly, in this study, we conducted an integrative analysis of TWASs and gene expression profiling to identify candidate genes associated with stroke. We aimed to combine the prediction approach of the TWAS with the experimental results of gene expression to provide novel insight into determination of the genes related to stroke. Gene ontology (GO) enrichment analysis was also performed to detect the pathways associated with stroke.

## Materials & Methods

### GWAS of stroke

The summary data for stoke were obtained from a large-scale GWAS meta-analysis conducted by the MEGASTROKE Consortium (Malik et al., 2018). All available stroke samples with published or unpublished GWAS data were combined in their essential study. We extracted the summary statistics restricted to European ancestry because the current available reference panels for TWAS were all established on European population. The final dataset was composed of 40,585 cases and 406,111 controls from 17 studies. Genotype imputation was conducted using 10,000 Genomes Project (1000G) phase 1v3 as the reference panel. After quality control, more than 8 million SNPs and indels with minor-allele frequencies greater than or equal to 0.01 were tested in the fixed-effects meta-analysis. The meta-analysis was carried out using METAL, and only SNPs passing the MEGASTROKE filters (number of cases >50% and imputation INFO score >5) were present in the final summary dataset.

### Performing TWAS on stroke GWAS dataset

We next performed a TWAS to identify significant expression-trait associations in stroke using FUSION software (http://gusevlab.org/projects/fusion/) (Gusev et al., 2016). Briefly, this approach took GWAS summary statistics on expression reference panels and linkage disequilibrium (LD) reference panels to identify significant expression-trait associations. The GWAS summary statistics of stroke were first converted into LD-score format, and SNPs were matched to the LD reference panel (EUR 1000 Genomes) to filter the part used for prediction. Summary-based imputation was then performed using three expression panels: RNA array data from the peripheral blood of 1,245 unrelated individuals from the Netherlands Twin Registry (NTR), RNA array data from the blood of 1,264 individuals from the Young Finns Study (YFS), and RNA-seq data from the adipose tissue of 563 individuals from the Metabolic Syndrome in Men study (METSIM) (Nuotio et al., 2014; Stancakova et al., 2012; Wright et al., 2014). We imputed the correction between predicted expression and stroke as a linear combination of GWAS *z*-scores (defined as *Z*) with expression weights (defined as *w*), adjusting with an SNP-correlation (LD) matrix D. Finally, the association between the cis-genetic component of expression and stroke was calculated according to the following formula as ZTWAS = *w*′*Z*∕(*w*′*Dw*)1∕2. In addition, the summarized association statistic might be inflated from by-chance quantitative trait loci (QTL) co-localization when the GWAS locus is highly significant and LD is extensive. To control for this, we further performed 5000 permutations which shuffled the expression weights and recalculated the empirical association statistic conditional on the GWAS effects at the locus. Genes with empirical permutation *P* < 0.05 were considered unlikely to be disturbed by QTLs.

### Gene expression profiling of stroke

The gene expression profiling data were obtained from a study by Krug et al. (2012). Krug et al. performed gene expression profile analysis in peripheral blood mononuclear cells from 20 patients who experienced ischemic stroke and 20 sex- and age-matched controls. All participants were European, and ischemic stroke patients were required to have suffered only one stroke episode, at least 6 months before blood collection. Expression microarrays were generated using Affymetrix Human Genome U133 Plus 2.0 Array and extensive quality control were performed in all steps. Multi-factor analysis of variance (ANOVA) was used to identify the differentially expressed genes among cases and controls, considering group, age, sex, the interactions among them, as well as the geographic origin of the participants and the scan-date of the microarrays as factors. The genes with a fold change >1.2 and an uncorrected *P* value <0.05 were considered differentially expressed as defined by Krug et al. (2012). After expression profile analysis, 709 probe sets (representing 580 genes) were identified by the gene expression profile analysis.

### Gene Ontology enrichment analysis

We combined TWAS results with gene expression profiling results to identify common genes for stroke. The candidate genes were then input into DAVID (https://david.ncifcrf.gov/home.jsp), a widely used tool for GO enrichment analysis. A primary enrichment *P* value was calculated for each GO term using DAVID. Significant GO terms were identified at *P* < 0.05.

## Results

**Figure 1:**
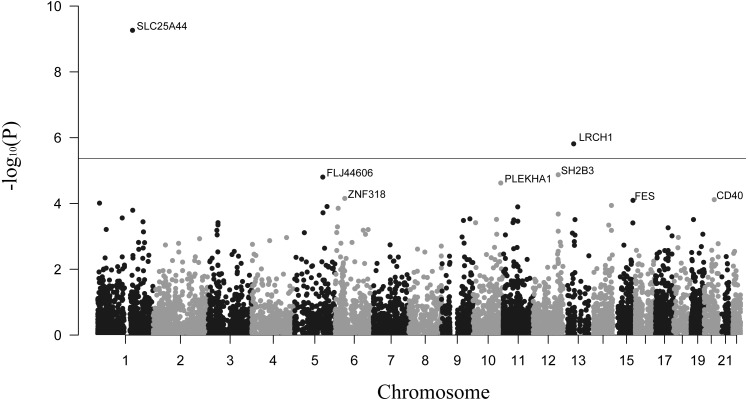
Manhattan plot showing genes associated with stroke. The dashed line shows the threshold for Bonferroni correction (*P* = 0.05/11,826).

The primary results for TWAS analysis were presented in Fig. 1. The distribution presented some inflation (*λ* = 1.13) of obtained versus expected results under the null hypothesis (Fig. S1). This was probably because the TWAS computed multiple models for each gene and outputted the statistics of the best performing model. In total, 11,826 tissue-specific features of gene expression were tested in the summary-based TWAS approach. After calculation, we identified 515 transcriptome-wide significant gene-stroke features (*P*_TWAS_ < 0.05 and *P*_permutation_ < 0.05) for 446 unique genes, including 58 genes that were significant in more than one panel (Table S1). The top 10 tissue-specific genes are summarized in Table 1. *SLC25A44* and *LRCH1* were the only two genes passing the Bonferroni correction threshold (*P* < 0.05/11,826) for all gene-stroke features, which implied that they were strongly associated with stroke. In order to cover more possible candidate genes, we also reported genes passing the transcriptome-wide significant threshold (*P* < 0.05) for integrative analysis.

**Table 1:** Top 10 significant tissue-specific genes identified by TWAS.

**Gene**	**Chromosome**	***Z*-score**	***P*_**TWAS**_**	***P*_**permutation**_**	**Tissue**
SLC25A44	1	6.2053	5.46E−10	0.00671	Adipose (METSIM)
LRCH1	13	−4.8058	1.54E−06	0.01153	Adipose (METSIM)
FLJ44606	5	4.3540	1.58E−05	0.01150	Peripheral blood (NTR)
PLEKHA1	10	−4.3171	2.39E−05	0.00499	Adipose (METSIM)
ZNF318	6	4.2252	7.04E−05	0.01974	Peripheral blood (NTR)
CD40	20	3.9748	7.67E−05	0.00376	Adipose (METSIM)
FES	15	−3.9545	8.06E−05	0.00515	Adipose (METSIM)
RERE	1	−3.9425	9.77E−05	0.00566	Peripheral blood (NTR)
SLC25A29	14	3.8962	1.15E−04	0.00100	Adipose (METSIM)
LARS	5	3.8558	1.24E−04	0.02459	Adipose (METSIM)

**Table 2:** List of overlapping candidate genes recognized by the integrative analysis of TWAS and gene expression profiling.

**Gene**	**CHR**	***P*_**TWAS**_**			***P*_**mRNA**_**
		**METSIM**	**NTR**	**YFS**	
CISD1	10	0.00357	/	/	0.0055
RHOU	1	/	/	0.00375	0.0276
TM6SF1	15	0.0136	/	0.00586	0.0452
TP53RK	20	/	/	0.00803	0.0205
CLDN5	22	/	/	0.00804	0.0008
NFKBIA	14	/	0.00957	/	0.0114
MRVI1	11	0.00997	/	/	0.0359
ZNF667-AS1	19	0.01025	/	/	0.0019
WDSUB1	2	/	0.01414	/	0.0153
WSB1	17	0.04353	/	0.01501	0.0348
TPM1	15	/	0.01590	/	0.0302
GOLGA8A	15	/	/	0.01704	0.0062
TIPARP	3	0.02357	/	/	0.0095
VRK2	2	/	/	0.02953	0.0211
TOR1AIP1	1	/	0.0339	/	0.0051
BARD1	2	0.03580	/	0.04980	0.0019
BAG5	14	/	0.03791	/	0.0410
VASH1	14	0.04918	/	0.03802	0.0239
PDIA6	2	/	/	0.04136	0.0031

We then compared transcriptome-wide significant genes identified by TWAS with the differentially expressed genes identified by gene expression profile analysis. Finally, 19 genes within 23 tissue-specific gene-stroke features were detected (Table 2). There were more shared genes (10/19) detected in the expression panel of the YFS with expression profiling. This may be because the expression profiling was performed using tissues similar to those used in YFS. In addition, four candidate genes showed transcriptome-wide significance in more than one panel.

GO enrichment analysis was conducted for the 19 candidate genes identified by the integrative analysis. We detected eight significant GO functional gene sets for stroke, including six biological process terms and two molecular function terms (Table 3). The most significant GO term for biological process was regulation of cell shape (*P* = 0.0059) while the most significant GO term for molecular function was the ubiquitin-protein transferase activity (*P* = 0.0336). Identification of these gene sets contributed to a better understanding of the pathogenesis of stroke.

**Table 3:** Gene Ontology enrichment analysis of the 27 overlapping candidate genes.

**Category**	**ID**	**Name**	***P*value**
GOTERM_BP_DIRECT	GO:0008360	Regulation of cell shape	0.0059
GOTERM_BP_DIRECT	GO:0060325	Face morphogenesis	0.0247
GOTERM_BP_DIRECT	GO:0032781	Positive regulation of ATPase activity	0.0256
GOTERM_MF_DIRECT	GO:0004842	Ubiquitin-protein transferase activity	0.0336
GOTERM_BP_DIRECT	GO:0016567	Protein ubiquitination	0.0350
GOTERM_MF_DIRECT	GO:0008092	Cytoskeletal protein binding	0.0418
GOTERM_BP_DIRECT	GO:0071407	Cellular response to organic cyclic compound	0.0481
GOTERM_BP_DIRECT	GO:0045732	Positive regulation of protein catabolic process	0.0489

## Discussion

In this study, we conducted an integrative analysis of TWAS and gene expression profiling data to identify candidate genes for stroke. In total, 19 causal genes were detected, and eight significant GO functional gene sets were identified. To the best of our knowledge, this was the first TWAS conducted on stroke, and our approach provided novel insights into understanding the genetic mechanism of stroke.

Our TWAS identified two genes, i.e., *SLC25A44* and *LRCH1*, that were significant for stroke after Bonferroni correction. *SLC25A44* is a known genetic risk factor for intracerebral hemorrhage (ICH), which is a common cause of stroke (Carpenter et al., 2016; Chen, Chang & Chen, 2018; Falcone et al., 2014). This gene was first mentioned as a risk gene for stroke in a GWAS conducted by the International Stroke Genetics Consortium (Woo et al., 2014). *SLC25A44* is a gene on locus 1q22 and encodes a mitochondrial carrier protein, suggesting that stroke may be associated with mitochondrial dysfunction. Moreover, a recent study found that *SLC25A44* was upregulated in obese patients throughout the arterial network based on rat models. Because the signal for *SLC25A44* was detected in adipose tissue, we suspected that this gene may have important roles in lipid metabolism. However, the biological mechanisms of *SLC25A44* remain unclear. *LRCH1* was also recognized as a risk gene for stroke in the GWAS included in our study. *LRCH1* was recently reported to be associated with atrioventricular nodal delay. Additionally, the regulatory factor for *LRCH1* was found to be active in multiple tissues, including the left ventricle, atherosclerotic aorta, atherosclerotic-lesion-free arteries, and blood. This suggested that *LRCH1* was strongly associated with cardiac mechanisms of stroke, such as large-artery atherosclerotic stroke, cardioembolic stroke, and small-vessel stroke. Recently, Xu et al. found that LRCH1 restrained T cell migration through binding partner to sequester *DOCK8* from *Cdc42* (Xu et al., 2017). T cell response after stroke is increasingly recognized in previous studies (Gill & Veltkamp, 2016). Evidence showed that antibody-mediated depletion of CD4(+), CD8(+) and gamma delta T cells could reduce infarct volume and improve functional outcome (Liesz et al., 2011; Mracsko et al., 2014; Shichita et al., 2009). Our study also showed that depressed expression of *LRCH1* was associated with stroke (Table 1). Thus, *LRCH1* might act a role in interfering T cell migration in stroke.

Of the 19 candidate genes identified by integrative analysis, *CISD1*, which encodes *CDGSH* iron sulfur domain 1, has been extensively studied. The main function of *CISD1* is modulation of mitochondrial iron uptake and respiratory capacity (Yuan et al., 2016). Loss of *CISD1* results in oxidative injury in the mitochondria, thereby affecting lipid and glucose metabolism. Furthermore, a recent clinical study showed that *CISD1* mRNA and protein levels were significantly decreased in subcutaneous and visceral adipose tissues of obese patients (Moreno-Navarrete et al., 2016). Moreover, *CISD1* is highly associated with adipose tissue dysfunction via regulation of mitochondrial dysfunction (Comas et al., 2018). Interestingly, *SLC25A44* also plays a role in mitochondrial function and is associated with lipid metabolism. Thus, we speculate that *SLC25A44* may have a pathophysiological mechanism similar to that of *CISD1*. Taken together, these findings suggested that stroke may be closely related to mitochondrial abnormalities.

*CLDN5* was another gene identified in this study and showed the strongest differential expression among all 19 candidate genes. *CLDN5* encodes an integral membrane protein that is a critical component of endothelial tight junctions (Jang et al., 2011). Previous studies have demonstrated that *CLDN5* plays important roles in the blood–brain barrier (Jiao et al., 2015). Additionally, an animal experiment showed that *CLDN5*-knockout mice show increased blood–brain barrier permeability (Nitta et al., 2003). In contrast, inhibiting *CLDN5* expression contributes to reduced brain edema and hemorrhagic transformation (Ishiguro et al., 2010). Increased blood–brain barrier permeability is a common pathological symptom after ICH, which could explain the differential expression of *CLDN5* in patients with stroke.

We also conducted GO enrichment analysis to determine the functions of the identified genes. We observed five GO gene sets for biological process, and the most significant GO term in this category was regulation of cell shape. According to a previous report, 92% of abnormal erythrocytes are present in smears of patients who have experienced thromboembolic ischemic stroke (Pretorius & Lipinski, 2013). Altered red blood cells form close interactions with abnormal fibrin fibers, typically resulting in a diseased clot. Moreover, protein modifications with ubiquitin are common responses for ischemia-reperfusion injury, accounting for the GO term of protein ubiquitination (Hochrainer, 2018). The regulation of ATPase activity may reflect the regulatory roles of mitochondria.

There were several limitations to our study. First, gene expression is a highly complex process, varying temporally and spatially in humans. The expression profiling in our study was performed only with peripheral blood mononuclear cells. Further work is needed to discover more candidate genes active in other tissues. Second, we extracted the summary statistics restricted to European ancestry as current available expression reference panels were all generated from European population. More samples from other ethnic groups should be collected to provide available reference panels for the entire multiethnic population. Third, the expression profiles analysis was performed with a relaxed criterion for determining differential expressed genes. Further work should be conducted with a larger samples and stricter criterion. Fourth, our study provided a novel method to determine candidate genes for stroke. However, functional studies are needed to determine the biological mechanisms underlying the roles of these genes in stroke.

## Conclusions

In summary, we conducted an integrative analysis of TWAS and gene expression profiling to determine the candidate genes for stroke. From this analysis, we identified 19 unique genes and eight corresponding functional gene sets for stoke. The annotation of some genes implied that mitochondria dysfunction may contribute to the cause of stroke. Some candidate genes also showed signals with regulation of cell shape and protein ubiquitination. Our study provided novel insights into the genetic mechanisms underlying stroke.

##  Supplemental Information

10.7717/peerj.7435/supp-1 Table S1 List of all candidate genes identified by TWAS for stroke (*P* value < 0.05)

10.7717/peerj.7435/supp-2 Figure S1 Quantile-quantile plot for the TWAS of stroke

## Additional Information and Declarations

### Data Availability

The following information was supplied regarding data availability: The GWAS summary results of stroke are available from the GWAS Catalog (https://www.ebi.ac.uk/gwas/), Publication ID 29531354. Tissue specific expression reference panels are publicly available on FUSION website (http://gusevlab.org/projects/fusion/). The data on gene expression profiling is available as a supplementary file named “10.1038_jcbfm.2012.24x2.doc” from this publication: https://journals.sagepub.com/doi/suppl/10.1038/jcbfm.2012.24.
