# Supplementary material for: Integrative analysis of transcriptome-wide association study and gene expression profiling identifies candidate genes associated with stroke
Source: PeerJ. 2019 Jul 29;7:e7435. doi: 10.7717/peerj.7435 (PMC6673425; doi:10.7717/peerj.7435)
Supplement: Table S1 [file peerj-07-7435-s001.docx]

**Table s1.** List of all candidate genes identified by TWAS for stroke (P value < 0.05)

| **Gene** | **CHR** | **Z-score** | ***P*_TWAS_** | ***P*_permutation_** | **Tissue** |
| --- | --- | --- | --- | --- | --- |
| SLC25A44 | 1 | 6.20532 | 5.46E-10 | 0.00671 | Adipose (METSIM) |
| LRCH1 | 13 | -4.8058 | 1.54E-06 | 0.01153 | Adipose (METSIM) |
| FLJ44606 | 5 | -4.3171 | 1.58E-05 | 0.0115 | Peripheral blood (NTR) |
| PLEKHA1 | 10 | 4.22524 | 2.39E-05 | 0.00499 | Adipose (METSIM) |
| ZNF318 | 6 | 3.97479 | 7.04E-05 | 0.01974 | Peripheral blood (NTR) |
| CD40 | 20 | -3.95451 | 7.67E-05 | 0.00376 | Adipose (METSIM) |
| FES | 15 | -3.9425 | 8.06E-05 | 0.00515 | Adipose (METSIM) |
| RERE | 1 | 3.89622 | 9.77E-05 | 0.00566 | Peripheral blood (NTR) |
| SLC25A29 | 14 | 3.85578 | 1.15E-04 | 0.001 | Adipose (METSIM) |
| LARS | 5 | -3.83719 | 1.24E-04 | 0.02459 | Adipose (METSIM) |
| CAPN1 | 11 | -3.83133 | 1.27E-04 | 0.00294 | Whole blood (YFS) |
| RNF182 | 6 | 3.80828 | 1.40E-04 | 0.00894 | Peripheral blood (NTR) |
| LARS | 5 | 3.7897 | 1.51E-04 | 0.04688 | Whole blood (YFS) |
| GNAI3 | 1 | -3.63672 | 2.76E-04 | 0.01449 | Whole blood (YFS) |
| NEK6 | 9 | -3.6215 | 2.93E-04 | 0.00426 | Peripheral blood (NTR) |
| SH3PXD2A | 10 | 3.6108 | 3.05E-04 | 0.03593 | Whole blood (YFS) |
| CKAP2 | 13 | 3.6068 | 3.10E-04 | 0.00793 | Peripheral blood (NTR) |
| NEK6 | 9 | -3.59771 | 3.21E-04 | 0.00282 | Whole blood (YFS) |
| PTCH1 | 9 | 3.59104 | 3.29E-04 | 0.002 | Whole blood (YFS) |
| LTBP3 | 11 | 3.57727 | 3.47E-04 | 0.0333 | Adipose (METSIM) |
| RERE | 1 | 3.57586 | 3.49E-04 | 0.02614 | Whole blood (YFS) |
| PPFIA4 | 1 | -3.56676 | 3.61E-04 | 0.01788 | Adipose (METSIM) |
| HSD17B12 | 11 | -3.55183 | 3.83E-04 | 0.0138 | Adipose (METSIM) |
| CCR3 | 3 | -3.55058 | 3.84E-04 | 0.0188 | Peripheral blood (NTR) |
| USP6NL | 10 | -3.54931 | 3.86E-04 | 0.00456 | Adipose (METSIM) |
| CIB1 | 15 | 3.5463 | 3.91E-04 | 0.00605 | Peripheral blood (NTR) |
| CKAP2 | 13 | 3.5277 | 4.19E-04 | 0.01041 | Adipose (METSIM) |
| CD40 | 20 | -3.5265 | 4.21E-04 | 0.00786 | Peripheral blood (NTR) |
| ZC3H14 | 14 | -3.5051 | 4.56E-04 | 0.00858 | Whole blood (YFS) |
| SYCP2L | 6 | -3.4736 | 5.14E-04 | 0.0002 | Adipose (METSIM) |
| BZRAP1 | 17 | 3.45571 | 5.49E-04 | 0.04412 | Whole blood (YFS) |
| FHL3 | 1 | -3.42351 | 6.18E-04 | 0 | Adipose (METSIM) |
| CKAP2 | 13 | 3.4229 | 6.20E-04 | 0.017 | Whole blood (YFS) |
| ZNF318 | 6 | -3.4219 | 6.22E-04 | 0.01987 | Whole blood (YFS) |
| STXBP5 | 6 | 3.42185 | 6.22E-04 | 0.04255 | Whole blood (YFS) |
| NCOA7 | 6 | -3.40954 | 6.51E-04 | 0.00605 | Whole blood (YFS) |
| ULK4 | 3 | -3.40681 | 6.57E-04 | 0.03593 | Whole blood (YFS) |
| WARS | 14 | -3.4065 | 6.58E-04 | 0.00421 | Peripheral blood (NTR) |
| CDK18 | 1 | -3.37771 | 7.31E-04 | 0.00578 | Adipose (METSIM) |
| RIOK1 | 6 | -3.36431 | 7.67E-04 | 0.00444 | Whole blood (YFS) |
| WARS | 14 | -3.3555 | 7.92E-04 | 0.0016 | Whole blood (YFS) |
| ELF1 | 13 | -3.3539 | 7.97E-04 | 0.00556 | Adipose (METSIM) |
| ZNF880 | 19 | -3.33209 | 8.62E-04 | 0.0018 | Peripheral blood (NTR) |
| AHI1 | 6 | -3.33056 | 8.67E-04 | 0.02294 | Peripheral blood (NTR) |
| SBF2 | 11 | -3.3187 | 9.04E-04 | 0.01353 | Peripheral blood (NTR) |
| SEC22C | 3 | 3.31865 | 9.05E-04 | 0.00828 | Whole blood (YFS) |
| VPS36 | 13 | 3.3154 | 9.15E-04 | 0.0305 | Whole blood (YFS) |
| PPFIA4 | 1 | -3.31198 | 9.26E-04 | 0.00962 | Whole blood (YFS) |
| CCR1 | 3 | 3.31094 | 9.30E-04 | 0.04633 | Whole blood (YFS) |
| ACOX1 | 17 | -3.29956 | 9.68E-04 | 0.00246 | Peripheral blood (NTR) |
| SEMA4D | 9 | 3.27756 | 1.05E-03 | 0.01121 | Whole blood (YFS) |
| NPC1 | 18 | -3.2695 | 1.08E-03 | 0.00446 | Adipose (METSIM) |
| PLRG1 | 4 | -3.26675 | 1.09E-03 | 0.0143 | Whole blood (YFS) |
| INO80D | 2 | 3.24456 | 1.18E-03 | 0.00865 | Whole blood (YFS) |
| RERE | 1 | 3.21055 | 1.32E-03 | 0.02649 | Adipose (METSIM) |
| ANXA3 | 4 | 3.20462 | 1.35E-03 | 0.01399 | Whole blood (YFS) |
| RCBTB1 | 13 | -3.1865 | 1.44E-03 | 0.02505 | Adipose (METSIM) |
| GCN1L1 | 12 | -3.17481 | 1.50E-03 | 0.04858 | Adipose (METSIM) |
| LAMC1 | 1 | -3.16903 | 1.53E-03 | 0.02277 | Whole blood (YFS) |
| COL12A1 | 6 | -3.1688 | 1.53E-03 | 0.03859 | Adipose (METSIM) |
| IRF6 | 1 | -3.16605 | 1.55E-03 | 0.02251 | Adipose (METSIM) |
| C9orf156 | 9 | -3.15 | 1.61E-03 | 0.0212 | Adipose (METSIM) |
| HFE | 6 | -3.152 | 1.62E-03 | 0.0018 | Adipose (METSIM) |
| CHCHD5 | 2 | 3.15083 | 1.63E-03 | 0.0029 | Whole blood (YFS) |
| RTEL1 | 20 | 3.14353 | 1.67E-03 | 0.03306 | Adipose (METSIM) |
| RNF4 | 4 | 3.13 | 1.75E-03 | 0.00608 | Adipose (METSIM) |
| POM121C | 7 | -3.12 | 1.81E-03 | 0.02844 | Adipose (METSIM) |
| ACYP2 | 2 | -3.117 | 1.83E-03 | 0.01575 | Peripheral blood (NTR) |
| ULK4 | 3 | -3.11676 | 1.83E-03 | 0.02128 | Adipose (METSIM) |
| CTD-3110H11.1 | 15 | -3.1137 | 1.85E-03 | 0.00609 | Adipose (METSIM) |
| KCNRG | 13 | -3.1083 | 1.88E-03 | 0.00853 | Adipose (METSIM) |
| EIF2B1 | 12 | 3.10069 | 1.93E-03 | 0.00644 | Peripheral blood (NTR) |
| MAF1 | 8 | -3.09269 | 1.98E-03 | 0.0199 | Whole blood (YFS) |
| SIX5 | 19 | -3.08315 | 2.05E-03 | 0.00434 | Peripheral blood (NTR) |
| NOC3L | 10 | -3.06687 | 2.16E-03 | 0.00509 | Adipose (METSIM) |
| ASRGL1 | 11 | 3.06496 | 2.18E-03 | 0.00497 | Whole blood (YFS) |
| ABHD3 | 18 | 3.0595 | 2.22E-03 | 0.01063 | Whole blood (YFS) |
| PLEKHA6 | 1 | 3.054 | 2.26E-03 | 0.01902 | Adipose (METSIM) |
| UBE2B | 5 | -3.0489 | 2.30E-03 | 0.01573 | Whole blood (YFS) |
| TACC1 | 8 | 3.0303 | 2.44E-03 | 0.028 | Peripheral blood (NTR) |
| NCF2 | 1 | -3.02558 | 2.48E-03 | 0.00862 | Peripheral blood (NTR) |
| TRPC4AP | 20 | 3.00557 | 2.65E-03 | 0.02592 | Whole blood (YFS) |
| ZNF500 | 16 | -3.004 | 2.66E-03 | 0.0006 | Adipose (METSIM) |
| DSP | 6 | -3.001 | 2.69E-03 | 0.02709 | Adipose (METSIM) |
| ZNF880 | 19 | -2.99498 | 2.74E-03 | 0.01407 | Adipose (METSIM) |
| ADPRH | 3 | 2.98174 | 2.87E-03 | 0 | Whole blood (YFS) |
| GGT1 | 22 | -2.98096 | 2.87E-03 | 0.0135 | Adipose (METSIM) |
| LAMC1 | 1 | -2.97906 | 2.89E-03 | 0.03288 | Peripheral blood (NTR) |
| PSD4 | 2 | -2.96735 | 3.00E-03 | 0.00969 | Peripheral blood (NTR) |
| TRAM1 | 8 | 2.96766 | 3.00E-03 | 0.0224 | Whole blood (YFS) |
| SREBF1 | 17 | 2.96316 | 3.04E-03 | 0.02685 | Adipose (METSIM) |
| AES | 19 | -2.958051 | 3.10E-03 | 0.0059 | Whole blood (YFS) |
| SDHAF1 | 19 | 2.943944 | 3.24E-03 | 0.0014 | Whole blood (YFS) |
| PRR11 | 17 | -2.94153 | 3.27E-03 | 0.04124 | Whole blood (YFS) |
| ZNF74 | 22 | -2.93919 | 3.29E-03 | 0 | Adipose (METSIM) |
| ELP5 | 17 | -2.92626 | 3.43E-03 | 0.00983 | Adipose (METSIM) |
| LLGL2 | 17 | 2.92456 | 3.45E-03 | 0.02444 | Adipose (METSIM) |
| CTSD | 11 | -2.92444 | 3.45E-03 | 0.00589 | Whole blood (YFS) |
| CORO1C | 12 | -2.92322 | 3.46E-03 | 0.00722 | Adipose (METSIM) |
| SLC2A9 | 4 | 2.92118 | 3.49E-03 | 0.03419 | Adipose (METSIM) |
| DPPA4 | 3 | 2.91381 | 3.57E-03 | 0.01306 | Adipose (METSIM) |
| CISD1 | 10 | -2.91357 | 3.57E-03 | 0.01602 | Adipose (METSIM) |
| PRDX3 | 10 | -2.9105 | 3.61E-03 | 0.0114 | Peripheral blood (NTR) |
| C14orf79 | 14 | 2.90404 | 3.68E-03 | 0.00744 | Adipose (METSIM) |
| RCBTB1 | 13 | -2.9017 | 3.71E-03 | 0.0391 | Whole blood (YFS) |
| RHOU | 1 | 2.89815 | 3.75E-03 | 0.00597 | Whole blood (YFS) |
| DCAF16 | 4 | -2.894 | 3.80E-03 | 0.00804 | Whole blood (YFS) |
| FCRL3 | 1 | 2.89302 | 3.82E-03 | 0.01796 | Peripheral blood (NTR) |
| AC138969.4 | 16 | -2.8872 | 3.89E-03 | 0.016 | Adipose (METSIM) |
| FCRL3 | 1 | 2.87908 | 3.99E-03 | 0.02113 | Whole blood (YFS) |
| MRAS | 3 | 2.87775 | 4.01E-03 | 0.00892 | Adipose (METSIM) |
| TMEM8B | 9 | 2.88 | 4.01E-03 | 0.0251 | Adipose (METSIM) |
| LINC00310 | 21 | 2.8689 | 4.12E-03 | 0.00777 | Adipose (METSIM) |
| PSMA5 | 1 | 2.86168 | 4.21E-03 | 0.02697 | Whole blood (YFS) |
| LIME1 | 20 | -2.86129 | 4.22E-03 | 0.02454 | Whole blood (YFS) |
| TRIM73 | 7 | -2.8613 | 4.22E-03 | 0.03448 | Adipose (METSIM) |
| KIAA0319 | 6 | -2.86135 | 4.22E-03 | 0.04651 | Peripheral blood (NTR) |
| LSMEM1 | 7 | -2.8572 | 4.27E-03 | 0.01002 | Adipose (METSIM) |
| CTD-2324F15.2 | 5 | -2.85225 | 4.34E-03 | 0.00621 | Adipose (METSIM) |
| ACAP1 | 17 | 2.84601 | 4.43E-03 | 0.02202 | Peripheral blood (NTR) |
| NUTM2B | 10 | 2.84393 | 4.46E-03 | 0.02586 | Adipose (METSIM) |
| CIB1 | 15 | 2.842983 | 4.47E-03 | 0.0223 | Whole blood (YFS) |
| RNF19B | 1 | 2.83937 | 4.52E-03 | 0.01166 | Peripheral blood (NTR) |
| CTDNEP1 | 17 | 2.83767 | 4.54E-03 | 0.0219 | Adipose (METSIM) |
| FCRL2 | 1 | -2.83564 | 4.57E-03 | 0.02073 | Peripheral blood (NTR) |
| FCRL3 | 1 | 2.83259 | 4.62E-03 | 0.02178 | Adipose (METSIM) |
| TIMM17A | 1 | -2.83001 | 4.65E-03 | 0.02264 | Adipose (METSIM) |
| TRIP11 | 14 | -2.8191 | 4.82E-03 | 0.02339 | Whole blood (YFS) |
| SCRN2 | 17 | -2.81185 | 4.93E-03 | 0.01455 | Adipose (METSIM) |
| C5orf22 | 5 | -2.8104 | 4.95E-03 | 0.0119 | Peripheral blood (NTR) |
| CWF19L2 | 11 | 2.80556 | 5.02E-03 | 0.01813 | Whole blood (YFS) |
| MAP2K2 | 19 | 2.803982 | 5.05E-03 | 0.04412 | Whole blood (YFS) |
| C10orf131 | 10 | -2.8024 | 5.07E-03 | 0.0236 | Peripheral blood (NTR) |
| CTNNBL1 | 20 | 2.80229 | 5.07E-03 | 0.02597 | Whole blood (YFS) |
| RBM18 | 9 | 2.79879 | 5.13E-03 | 0.0223 | Whole blood (YFS) |
| FAM154B | 15 | -2.792887 | 5.22E-03 | 0.0124 | Whole blood (YFS) |
| DSP | 6 | -2.792 | 5.24E-03 | 0.02348 | Peripheral blood (NTR) |
| CD40 | 20 | -2.78395 | 5.37E-03 | 0.03738 | Whole blood (YFS) |
| GGCX | 2 | -2.78064 | 5.43E-03 | 0.00422 | Whole blood (YFS) |
| ZFYVE28 | 4 | -2.77838 | 5.46E-03 | 0.04332 | Whole blood (YFS) |
| ZNF804A | 2 | 2.77775 | 5.47E-03 | 0.01732 | Peripheral blood (NTR) |
| RPL36 | 19 | -2.774717 | 5.53E-03 | 0.00717 | Whole blood (YFS) |
| ATG7 | 3 | 2.77464 | 5.53E-03 | 0.01327 | Whole blood (YFS) |
| GATAD1 | 7 | 2.76902 | 5.62E-03 | 0 | Whole blood (YFS) |
| MPHOSPH6 | 16 | 2.7655 | 5.68E-03 | 0.00782 | Whole blood (YFS) |
| STEAP2 | 7 | -2.7639 | 5.71E-03 | 0.00654 | Adipose (METSIM) |
| LEFTY2 | 1 | -2.75878 | 5.80E-03 | 0.00558 | Adipose (METSIM) |
| SLC18A2 | 10 | -2.75741 | 5.83E-03 | 0.00729 | Adipose (METSIM) |
| TM6SF1 | 15 | 2.755378 | 5.86E-03 | 0.0191 | Whole blood (YFS) |
| C6ORF165 | 6 | -2.7523 | 5.92E-03 | 0.02062 | Adipose (METSIM) |
| TTLL3 | 3 | 2.75218 | 5.92E-03 | 0.0121 | Adipose (METSIM) |
| SNCAIP | 5 | 2.75148 | 5.93E-03 | 0.02661 | Adipose (METSIM) |
| MOCS2 | 5 | -2.74858 | 5.99E-03 | 0.01035 | Adipose (METSIM) |
| SPDL1 | 5 | -2.74574 | 6.04E-03 | 0.02335 | Adipose (METSIM) |
| CCDC115 | 2 | 2.74465 | 6.06E-03 | 0.00659 | Whole blood (YFS) |
| ZNF787 | 19 | 2.739622 | 6.15E-03 | 0.01558 | Whole blood (YFS) |
| MED27 | 9 | 2.73926 | 6.16E-03 | 0.02069 | Whole blood (YFS) |
| TOMM5 | 9 | 2.7355 | 6.23E-03 | 0.01353 | Peripheral blood (NTR) |
| MAN1B1 | 9 | -2.73 | 6.26E-03 | 0.0008 | Adipose (METSIM) |
| PIM1 | 6 | -2.72914 | 6.35E-03 | 0.02553 | Whole blood (YFS) |
| PFN2 | 3 | -2.71511 | 6.63E-03 | 0.01 | Peripheral blood (NTR) |
| BZW2 | 7 | 2.71377 | 6.65E-03 | 0.00841 | Whole blood (YFS) |
| ENPP3 | 6 | -2.7126 | 6.68E-03 | 0.01293 | Adipose (METSIM) |
| FHL3 | 1 | -2.71117 | 6.71E-03 | 0.00526 | Whole blood (YFS) |
| WRB | 21 | 2.709 | 6.74E-03 | 0.0135 | Peripheral blood (NTR) |
| KIAA0020 | 9 | 2.707 | 6.79E-03 | 0.02597 | Whole blood (YFS) |
| NOMO3 | 16 | -2.7064 | 6.80E-03 | 0.014 | Adipose (METSIM) |
| NOC3L | 10 | -2.705 | 6.83E-03 | 0.0453 | Peripheral blood (NTR) |
| SH3BGR | 21 | 2.704 | 6.85E-03 | 0.00762 | Adipose (METSIM) |
| MAPK9 | 5 | -2.7019 | 6.89E-03 | 0.02542 | Whole blood (YFS) |
| ZNF500 | 16 | -2.69572 | 7.02E-03 | 0.00372 | Whole blood (YFS) |
| MPEG1 | 11 | 2.69538 | 7.03E-03 | 0.00585 | Whole blood (YFS) |
| KCTD20 | 6 | -2.69384 | 7.06E-03 | 0.01849 | Whole blood (YFS) |
| C15orf40 | 15 | -2.6933 | 7.08E-03 | 0.0239 | Adipose (METSIM) |
| TSHZ2 | 20 | 2.69245 | 7.09E-03 | 0.02948 | Whole blood (YFS) |
| C16orf75 | 16 | -2.69014 | 7.14E-03 | 0.02048 | Peripheral blood (NTR) |
| CENPN | 16 | 2.68882 | 7.17E-03 | 0.03859 | Whole blood (YFS) |
| FHL3 | 1 | -2.68544 | 7.24E-03 | 0.0046 | Peripheral blood (NTR) |
| INO80E | 16 | 2.67673 | 7.43E-03 | 0.00569 | Peripheral blood (NTR) |
| MPHOSPH6 | 16 | 2.6764 | 7.44E-03 | 0.01125 | Peripheral blood (NTR) |
| WARS2 | 1 | -2.67488 | 7.48E-03 | 0.01775 | Adipose (METSIM) |
| ASRGL1 | 11 | 2.6718 | 7.54E-03 | 0.02376 | Peripheral blood (NTR) |
| AS3MT | 10 | -2.6697 | 7.59E-03 | 0.0451 | Peripheral blood (NTR) |
| RELL2 | 5 | 2.669 | 7.61E-03 | 0.01017 | Whole blood (YFS) |
| WRB | 21 | 2.6676 | 7.64E-03 | 0.01407 | Adipose (METSIM) |
| HEATR4 | 14 | 2.6647 | 7.71E-03 | 0.04082 | Peripheral blood (NTR) |
| PTPRJ | 11 | 2.6623 | 7.76E-03 | 0.003 | Peripheral blood (NTR) |
| TOMM5 | 9 | -2.66201 | 7.77E-03 | 0.00811 | Whole blood (YFS) |
| SLFN11 | 17 | 2.66097 | 7.79E-03 | 0.00993 | Whole blood (YFS) |
| MSH3 | 5 | 2.6577 | 7.87E-03 | 0.01083 | Adipose (METSIM) |
| CWF19L2 | 11 | 2.65586 | 7.91E-03 | 0.0269 | Adipose (METSIM) |
| TP53RK | 20 | -2.65082 | 8.03E-03 | 0.01471 | Whole blood (YFS) |
| CLDN5 | 22 | 2.6503 | 8.04E-03 | 0.0178 | Whole blood (YFS) |
| CTSF | 11 | -2.64654 | 8.13E-03 | 0.00481 | Whole blood (YFS) |
| CTB-12O2.1 | 5 | 2.64394 | 8.20E-03 | 0.04651 | Adipose (METSIM) |
| NOC4L | 12 | 2.64371 | 8.20E-03 | 0.00517 | Adipose (METSIM) |
| PDE8B | 5 | -2.6436 | 8.20E-03 | 0.0486 | Peripheral blood (NTR) |
| PUS7 | 7 | 2.64308 | 8.22E-03 | 0.0016 | Whole blood (YFS) |
| RIOK1 | 6 | -2.631 | 8.51E-03 | 0.02721 | Adipose (METSIM) |
| EIF6 | 20 | -2.63044 | 8.53E-03 | 0.03922 | Whole blood (YFS) |
| ZBTB9 | 6 | 2.62997 | 8.54E-03 | 0.0113 | Whole blood (YFS) |
| ATOX1 | 5 | -2.6286 | 8.58E-03 | 0.01562 | Whole blood (YFS) |
| NTN4 | 12 | 2.62833 | 8.58E-03 | 0.01483 | Adipose (METSIM) |
| PSMB3 | 17 | 2.62795 | 8.59E-03 | 0.01712 | Adipose (METSIM) |
| TBK1 | 12 | 2.62245 | 8.73E-03 | 0.0018 | Whole blood (YFS) |
| ACAD11 | 3 | 2.62139 | 8.76E-03 | 0.03 | Adipose (METSIM) |
| BEND7 | 10 | 2.61661 | 8.88E-03 | 0.03727 | Whole blood (YFS) |
| VPS13C | 15 | -2.6159 | 8.90E-03 | 0.03458 | Adipose (METSIM) |
| LIPA | 10 | 2.6102 | 9.05E-03 | 0.0157 | Peripheral blood (NTR) |
| PRDX3 | 10 | -2.60875 | 9.09E-03 | 0.03987 | Adipose (METSIM) |
| ACOX1 | 17 | -2.60349 | 9.23E-03 | 0.02771 | Whole blood (YFS) |
| SF3A3 | 1 | 2.60293 | 9.24E-03 | 0.00999 | Peripheral blood (NTR) |
| BOD1 | 5 | 2.59991 | 9.33E-03 | 0.03582 | Adipose (METSIM) |
| NOC3L | 10 | -2.5989 | 9.35E-03 | 0.02697 | Whole blood (YFS) |
| ANXA3 | 4 | -2.5976 | 9.39E-03 | 0.03183 | Adipose (METSIM) |
| TDRD9 | 14 | -2.5972 | 9.40E-03 | 0.0002 | Peripheral blood (NTR) |
| SF3A3 | 1 | -2.59236 | 9.53E-03 | 0.00733 | Whole blood (YFS) |
| ME2 | 18 | 2.591 | 9.57E-03 | 0.00638 | Whole blood (YFS) |
| NFKBIA | 14 | 2.5909 | 9.57E-03 | 0.0427 | Peripheral blood (NTR) |
| TBK1 | 12 | 2.58604 | 9.71E-03 | 0.0018 | Adipose (METSIM) |
| UROS | 10 | 2.58526 | 9.73E-03 | 0.00366 | Adipose (METSIM) |
| SLC26A8 | 6 | 2.585 | 9.74E-03 | 0.04196 | Adipose (METSIM) |
| ADORA3 | 1 | -2.58467 | 9.75E-03 | 0.01332 | Whole blood (YFS) |
| ATP8A1 | 4 | -2.58419 | 9.76E-03 | 0.01143 | Adipose (METSIM) |
| ELMSAN1 | 14 | -2.5821 | 9.82E-03 | 0.04317 | Adipose (METSIM) |
| QSOX1 | 1 | -2.57778 | 9.94E-03 | 0.0375 | Whole blood (YFS) |
| MRVI1 | 11 | 2.57701 | 9.97E-03 | 0.0264 | Adipose (METSIM) |
| ZNF667-AS1 | 19 | 2.5673 | 1.03E-02 | 0.0354 | Adipose (METSIM) |
| LRRC16A | 6 | -2.56617 | 1.03E-02 | 0.02791 | Peripheral blood (NTR) |
| TERF2 | 16 | 2.5656 | 1.03E-02 | 0.04152 | Whole blood (YFS) |
| WRB | 21 | 2.56649 | 1.03E-02 | 0.0453 | Whole blood (YFS) |
| PTCH1 | 9 | 2.5649 | 1.03E-02 | 0.01875 | Peripheral blood (NTR) |
| RHCE | 1 | -2.56208 | 1.04E-02 | 0.00844 | Adipose (METSIM) |
| SPATA7 | 14 | 2.5483 | 1.08E-02 | 0.04781 | Whole blood (YFS) |
| ACOT4 | 14 | 2.5446 | 1.09E-02 | 0.04938 | Whole blood (YFS) |
| TMEM106B | 7 | 2.54292 | 1.10E-02 | 0.01204 | Whole blood (YFS) |
| IMP4 | 2 | 2.54267 | 1.10E-02 | 0.00846 | Whole blood (YFS) |
| DHX34 | 19 | -2.54196 | 1.10E-02 | 0.04348 | Adipose (METSIM) |
| RP11-1017G21.6 | 14 | 2.54042 | 1.11E-02 | 0.01619 | Adipose (METSIM) |
| RNF185 | 22 | 2.53984 | 1.11E-02 | 0.0423 | Adipose (METSIM) |
| NUBP1 | 16 | 2.53966 | 1.11E-02 | 0.01178 | Whole blood (YFS) |
| RBBP5 | 1 | -2.54081 | 1.11E-02 | 0.01213 | Peripheral blood (NTR) |
| LRRC16A | 6 | -2.5385 | 1.11E-02 | 0.03582 | Whole blood (YFS) |
| KLHL35 | 11 | -2.53827 | 1.11E-02 | 0.04743 | Whole blood (YFS) |
| BAG4 | 8 | -2.5276 | 1.15E-02 | 0.0203 | Peripheral blood (NTR) |
| ROMO1 | 20 | -2.52647 | 1.15E-02 | 0.00423 | Whole blood (YFS) |
| DCSTAMP | 8 | 2.52481 | 1.16E-02 | 0.00895 | Adipose (METSIM) |
| TMEM56 | 1 | 2.52264 | 1.16E-02 | 0.01078 | Adipose (METSIM) |
| ADPRH | 3 | 2.52371 | 1.16E-02 | 0.0025 | Adipose (METSIM) |
| SLC35F6 | 2 | 2.5212 | 1.17E-02 | 0.00901 | Adipose (METSIM) |
| TNNT3 | 11 | -2.51813 | 1.18E-02 | 0.00903 | Whole blood (YFS) |
| TMC4 | 19 | -2.515385 | 1.19E-02 | 0.00773 | Whole blood (YFS) |
| TF | 3 | 2.51487 | 1.19E-02 | 0.01469 | Adipose (METSIM) |
| FES | 15 | -2.51489 | 1.19E-02 | 0.0219 | Whole blood (YFS) |
| UBE2M | 19 | -2.513679 | 1.19E-02 | 0.01633 | Whole blood (YFS) |
| UROS | 10 | 2.51121 | 1.20E-02 | 0.00285 | Whole blood (YFS) |
| CLIP3 | 19 | -2.511103 | 1.20E-02 | 0.01193 | Whole blood (YFS) |
| GPR153 | 1 | 2.50827 | 1.21E-02 | 0.02824 | Adipose (METSIM) |
| MAP3K1 | 5 | -2.5077 | 1.22E-02 | 0.01166 | Whole blood (YFS) |
| TRDV3 | 14 | -2.5003 | 1.24E-02 | 0.00852 | Peripheral blood (NTR) |
| ZFP1 | 16 | 2.50036 | 1.24E-02 | 0.02264 | Whole blood (YFS) |
| ENTPD4 | 8 | 2.49913 | 1.25E-02 | 0.03038 | Adipose (METSIM) |
| SCARB1 | 12 | -2.4955 | 1.26E-02 | 0.00657 | Whole blood (YFS) |
| LIPA | 10 | 2.49535 | 1.26E-02 | 0.0338 | Adipose (METSIM) |
| BTBD7 | 14 | -2.4938 | 1.26E-02 | 0.01562 | Whole blood (YFS) |
| ATF5 | 19 | -2.49033 | 1.28E-02 | 0.03261 | Adipose (METSIM) |
| ST6GALNAC6 | 9 | -2.48974 | 1.28E-02 | 0.00431 | Whole blood (YFS) |
| CANX | 5 | -2.48635 | 1.29E-02 | 0.04444 | Adipose (METSIM) |
| RP11-397A16.2 | 18 | 2.4853 | 1.29E-02 | 0.01441 | Adipose (METSIM) |
| ZNF497 | 19 | 2.48233 | 1.31E-02 | 0.01818 | Peripheral blood (NTR) |
| PIAS4 | 19 | -2.48198 | 1.31E-02 | 0.02464 | Peripheral blood (NTR) |
| NELFCD | 20 | -2.47366 | 1.34E-02 | 0.00935 | Adipose (METSIM) |
| USP8 | 15 | -2.4687 | 1.36E-02 | 0.0355 | Peripheral blood (NTR) |
| RNF141 | 11 | 2.46774 | 1.36E-02 | 0.04979 | Whole blood (YFS) |
| TM6SF1 | 15 | 2.4678 | 1.36E-02 | 0.03715 | Adipose (METSIM) |
| ACPP | 3 | -2.46656 | 1.36E-02 | 0.0134 | Peripheral blood (NTR) |
| MARCH7 | 2 | 2.46629 | 1.37E-02 | 0.01722 | Whole blood (YFS) |
| C3orf14 | 3 | -2.46602 | 1.37E-02 | 0.01098 | Adipose (METSIM) |
| PSMB8 | 6 | 2.4614 | 1.38E-02 | 0.036 | Peripheral blood (NTR) |
| NUP93 | 16 | -2.45914 | 1.39E-02 | 0.02667 | Whole blood (YFS) |
| FAM154B | 15 | -2.457 | 1.40E-02 | 0.0117 | Adipose (METSIM) |
| MGST2 | 4 | 2.45385 | 1.41E-02 | 0.0136 | Peripheral blood (NTR) |
| WDSUB1 | 2 | 2.45376 | 1.41E-02 | 0.02709 | Peripheral blood (NTR) |
| FAM50B | 6 | -2.4523 | 1.42E-02 | 0.00955 | Whole blood (YFS) |
| KBTBD7 | 13 | 2.4447 | 1.45E-02 | 0.02094 | Peripheral blood (NTR) |
| UBE2L3 | 22 | 2.44433 | 1.45E-02 | 0.0139 | Adipose (METSIM) |
| ASIC3 | 7 | -2.4419 | 1.46E-02 | 0.03488 | Adipose (METSIM) |
| RP11-266L9.5 | 16 | -2.4405 | 1.47E-02 | 0.0116 | Adipose (METSIM) |
| SMIM20 | 4 | 2.4402 | 1.47E-02 | 0.01678 | Adipose (METSIM) |
| SELK | 3 | 2.43922 | 1.47E-02 | 0.02464 | Adipose (METSIM) |
| DDX31 | 9 | -2.44 | 1.47E-02 | 0.00913 | Adipose (METSIM) |
| WSB1 | 17 | 2.43208 | 1.50E-02 | 0.01182 | Whole blood (YFS) |
| AK1 | 9 | -2.43 | 1.51E-02 | 0.00437 | Adipose (METSIM) |
| MARCH6 | 5 | 2.4294 | 1.51E-02 | 0.03429 | Whole blood (YFS) |
| IMPA2 | 18 | 2.4277 | 1.52E-02 | 0.0194 | Peripheral blood (NTR) |
| ST6GAL1 | 3 | 2.42686 | 1.52E-02 | 0.03604 | Whole blood (YFS) |
| PTPLAD2 | 9 | -2.42 | 1.54E-02 | 0.04348 | Adipose (METSIM) |
| SEL1L3 | 4 | 2.42064 | 1.55E-02 | 0.0338 | Adipose (METSIM) |
| MDGA1 | 6 | -2.41679 | 1.57E-02 | 0.03499 | Peripheral blood (NTR) |
| NUCB2 | 11 | -2.41501 | 1.57E-02 | 0.0232 | Adipose (METSIM) |
| FAM57A | 17 | -2.41482 | 1.57E-02 | 0.01729 | Adipose (METSIM) |
| KREMEN1 | 22 | 2.4144 | 1.58E-02 | 0.048 | Whole blood (YFS) |
| MCFD2 | 2 | -2.41295 | 1.58E-02 | 0.02277 | Adipose (METSIM) |
| TPM1 | 15 | -2.4112 | 1.59E-02 | 0.03217 | Peripheral blood (NTR) |
| TDRD9 | 14 | -2.4088 | 1.60E-02 | 0.01309 | Whole blood (YFS) |
| SIGLEC5 | 19 | 2.408588 | 1.60E-02 | 0.02626 | Whole blood (YFS) |
| LIMK2 | 22 | 2.406 | 1.61E-02 | 0.0147 | Whole blood (YFS) |
| NPRL3 | 16 | 2.4048 | 1.62E-02 | 0.0228 | Adipose (METSIM) |
| AP003733.1 | 11 | -2.40295 | 1.63E-02 | 0.0423 | Adipose (METSIM) |
| ITFG3 | 16 | 2.4016 | 1.63E-02 | 0.044 | Adipose (METSIM) |
| COL4A4 | 2 | 2.40083 | 1.64E-02 | 0.02586 | Whole blood (YFS) |
| PWWP2B | 10 | -2.39358 | 1.67E-02 | 0.02956 | Whole blood (YFS) |
| CDA | 1 | 2.38812 | 1.69E-02 | 0.00824 | Adipose (METSIM) |
| SF3A3 | 1 | -2.38682 | 1.70E-02 | 0.00507 | Adipose (METSIM) |
| UBAP1L | 15 | -2.3868 | 1.70E-02 | 0.01754 | Adipose (METSIM) |
| AF127936.7 | 21 | -2.386 | 1.70E-02 | 0.01427 | Adipose (METSIM) |
| GOLGA8A | 15 | 2.385883 | 1.70E-02 | 0.0212 | Whole blood (YFS) |
| LRRC18 | 10 | -2.3847 | 1.71E-02 | 0.0236 | Peripheral blood (NTR) |
| HLA-DOB | 6 | 2.3849 | 1.71E-02 | 0.0449 | Peripheral blood (NTR) |
| ARMC1 | 8 | -2.38307 | 1.72E-02 | 0.0311 | Whole blood (YFS) |
| KLHL23 | 2 | 2.37827 | 1.74E-02 | 0.02414 | Whole blood (YFS) |
| PSD4 | 2 | -2.37885 | 1.74E-02 | 0.04528 | Adipose (METSIM) |
| ZNF101 | 19 | -2.378026 | 1.74E-02 | 0.04167 | Whole blood (YFS) |
| GLB1L | 2 | -2.377 | 1.75E-02 | 0.01893 | Adipose (METSIM) |
| BFSP1 | 20 | -2.3756 | 1.75E-02 | 0.04762 | Peripheral blood (NTR) |
| ZFAND1 | 8 | 2.36883 | 1.78E-02 | 0.02449 | Adipose (METSIM) |
| ANKDD1B | 5 | 2.36449 | 1.81E-02 | 0.00899 | Adipose (METSIM) |
| RNF138 | 18 | -2.3563 | 1.85E-02 | 0.0315 | Whole blood (YFS) |
| CBL | 11 | -2.34869 | 1.88E-02 | 0.01525 | Whole blood (YFS) |
| DHX32 | 10 | 2.3468 | 1.89E-02 | 0.0194 | Peripheral blood (NTR) |
| WDR60 | 7 | 2.34542 | 1.90E-02 | 0.02626 | Whole blood (YFS) |
| NEFH | 22 | -2.3419 | 1.92E-02 | 0.0208 | Whole blood (YFS) |
| CPM | 12 | -2.33918 | 1.93E-02 | 0.03871 | Peripheral blood (NTR) |
| SERP2 | 13 | 2.3381 | 1.94E-02 | 0.0298 | Whole blood (YFS) |
| SH2D4B | 10 | 2.33428 | 1.96E-02 | 0.04839 | Adipose (METSIM) |
| TPPP | 5 | -2.33033 | 1.98E-02 | 0.03191 | Adipose (METSIM) |
| DNAJA4 | 15 | 2.328806 | 1.99E-02 | 0.0458 | Whole blood (YFS) |
| STAM2 | 2 | -2.32879 | 1.99E-02 | 0.04598 | Adipose (METSIM) |
| SHPRH | 6 | 2.32808 | 1.99E-02 | 0.01297 | Whole blood (YFS) |
| BCCIP | 10 | 2.3256 | 2.00E-02 | 0.0306 | Peripheral blood (NTR) |
| TCTN2 | 12 | 2.32451 | 2.01E-02 | 0.04959 | Adipose (METSIM) |
| HSPA12A | 10 | -2.32326 | 2.02E-02 | 0.02116 | Adipose (METSIM) |
| FAM129A | 1 | 2.31856 | 2.04E-02 | 0.04444 | Whole blood (YFS) |
| KIAA1826 | 11 | -2.3175 | 2.05E-02 | 0.001 | Peripheral blood (NTR) |
| LUZP1 | 1 | -2.31632 | 2.05E-02 | 0.02405 | Whole blood (YFS) |
| ABCD4 | 14 | 2.31615 | 2.06E-02 | 0.0241 | Adipose (METSIM) |
| MED10 | 5 | 2.31201 | 2.08E-02 | 0.01878 | Adipose (METSIM) |
| DNAH6 | 2 | -2.31121 | 2.08E-02 | 0.03053 | Adipose (METSIM) |
| GNPDA2 | 4 | 2.30942 | 2.09E-02 | 0.02542 | Adipose (METSIM) |
| CCDC146 | 7 | -2.30698 | 2.11E-02 | 0.03659 | Whole blood (YFS) |
| SLFN13 | 17 | -2.30606 | 2.11E-02 | 0.03261 | Whole blood (YFS) |
| PRPF38A | 1 | -2.30168 | 2.14E-02 | 0.01905 | Whole blood (YFS) |
| TCEB3 | 1 | 2.29856 | 2.15E-02 | 0.02637 | Whole blood (YFS) |
| PLXDC1 | 17 | 2.29814 | 2.16E-02 | 0.02559 | Whole blood (YFS) |
| VPREB3 | 22 | -2.29795 | 2.16E-02 | 0.0365 | Adipose (METSIM) |
| RHD | 1 | 2.28992 | 2.20E-02 | 0.02532 | Peripheral blood (NTR) |
| FAM173B | 5 | -2.2874 | 2.22E-02 | 0.03478 | Whole blood (YFS) |
| POLR3K | 16 | -2.28444 | 2.24E-02 | 0.04898 | Peripheral blood (NTR) |
| IWS1 | 2 | -2.28404 | 2.24E-02 | 0.0315 | Whole blood (YFS) |
| EIF4E3 | 3 | -2.28294 | 2.24E-02 | 0.01283 | Whole blood (YFS) |
| COMMD9 | 11 | 2.28183 | 2.25E-02 | 0.0354 | Adipose (METSIM) |
| PICALM | 11 | 2.2808 | 2.26E-02 | 0.03834 | Peripheral blood (NTR) |
| RIPK1 | 6 | 2.2779 | 2.27E-02 | 0.01434 | Adipose (METSIM) |
| CCDC104 | 2 | 2.27725 | 2.28E-02 | 0.02317 | Adipose (METSIM) |
| CNOT6L | 4 | -2.27566 | 2.29E-02 | 0.03859 | Adipose (METSIM) |
| DHX32 | 10 | 2.2753 | 2.29E-02 | 0.04762 | Whole blood (YFS) |
| PHYH | 10 | -2.27495 | 2.29E-02 | 0.04598 | Adipose (METSIM) |
| HACE1 | 6 | 2.2751 | 2.29E-02 | 0.048 | Adipose (METSIM) |
| DUSP16 | 12 | -2.27401 | 2.30E-02 | 0.03438 | Whole blood (YFS) |
| SLFN13 | 17 | -2.27186 | 2.31E-02 | 0.01076 | Peripheral blood (NTR) |
| ELL | 19 | -2.27084 | 2.32E-02 | 0.02771 | Adipose (METSIM) |
| LPIN2 | 18 | -2.2705 | 2.32E-02 | 0.03774 | Adipose (METSIM) |
| UBR3 | 2 | 2.26981 | 2.32E-02 | 0.04167 | Whole blood (YFS) |
| ATP6V1G2 | 6 | 2.26854 | 2.33E-02 | 0.0216 | Whole blood (YFS) |
| PHF19 | 9 | -2.26841 | 2.33E-02 | 0.02143 | Whole blood (YFS) |
| TIPARP | 3 | -2.26413 | 2.36E-02 | 0.02027 | Adipose (METSIM) |
| SLFN13 | 17 | -2.2616 | 2.37E-02 | 0.0131 | Adipose (METSIM) |
| GIGYF1 | 7 | -2.2573 | 2.40E-02 | 0.03704 | Adipose (METSIM) |
| MTL5 | 11 | -2.2544 | 2.42E-02 | 0.03235 | Whole blood (YFS) |
| UBE2R2 | 9 | -2.25368 | 2.42E-02 | 0.01881 | Whole blood (YFS) |
| KRTCAP3 | 2 | -2.25333 | 2.42E-02 | 0.0004 | Whole blood (YFS) |
| RP11-543C4.1 | 14 | 2.2516 | 2.43E-02 | 0.04598 | Adipose (METSIM) |
| HLA-DPB1 | 6 | -2.2509 | 2.44E-02 | 0.0349 | Adipose (METSIM) |
| PRKCB | 16 | -2.2508 | 2.44E-02 | 0.0233 | Adipose (METSIM) |
| GIGYF1 | 7 | -2.25054 | 2.44E-02 | 0.02198 | Whole blood (YFS) |
| GLOD4 | 17 | -2.24566 | 2.47E-02 | 0.03593 | Whole blood (YFS) |
| C21orf89 | 21 | -2.24 | 2.51E-02 | 0.0478 | Peripheral blood (NTR) |
| RAPGEF3 | 12 | -2.23836 | 2.52E-02 | 0.02317 | Adipose (METSIM) |
| UBN1 | 16 | 2.2381 | 2.52E-02 | 0.013 | Adipose (METSIM) |
| DHX38 | 16 | 2.2364 | 2.53E-02 | 0.036 | Adipose (METSIM) |
| ATP2B4 | 1 | -2.23254 | 2.56E-02 | 0.0219 | Adipose (METSIM) |
| CCDC115 | 2 | 2.23251 | 2.56E-02 | 0.03571 | Adipose (METSIM) |
| RCN3 | 19 | 2.230936 | 2.57E-02 | 0.0262 | Whole blood (YFS) |
| KIAA1147 | 7 | -2.23113 | 2.57E-02 | 0.0291 | Peripheral blood (NTR) |
| AK1 | 9 | -2.22792 | 2.59E-02 | 0.01644 | Whole blood (YFS) |
| TRIM24 | 7 | -2.2265 | 2.60E-02 | 0.02784 | Adipose (METSIM) |
| RYBP | 3 | 2.22459 | 2.61E-02 | 0.0368 | Peripheral blood (NTR) |
| MGST2 | 4 | 2.22341 | 2.62E-02 | 0.032 | Adipose (METSIM) |
| RHD | 1 | 2.22187 | 2.63E-02 | 0.04364 | Adipose (METSIM) |
| PROCA1 | 17 | 2.22173 | 2.63E-02 | 0.0458 | Whole blood (YFS) |
| NUP88 | 17 | 2.22172 | 2.63E-02 | 0.03738 | Whole blood (YFS) |
| FGL2 | 7 | -2.21799 | 2.66E-02 | 0.0185 | Peripheral blood (NTR) |
| NUP88 | 17 | 2.21364 | 2.69E-02 | 0.0297 | Peripheral blood (NTR) |
| TTC3 | 21 | -2.20965 | 2.71E-02 | 0.043 | Whole blood (YFS) |
| CCDC116 | 22 | -2.20606 | 2.74E-02 | 0.0325 | Adipose (METSIM) |
| CAMK2G | 10 | 2.2042 | 2.75E-02 | 0.02691 | Whole blood (YFS) |
| RP11-381K20.5 | 5 | -2.20418 | 2.75E-02 | 0.0327 | Adipose (METSIM) |
| HLA-DOB | 6 | 2.2041 | 2.75E-02 | 0.0284 | Adipose (METSIM) |
| MGST2 | 4 | 2.19825 | 2.79E-02 | 0.0367 | Whole blood (YFS) |
| UCHL5 | 1 | -2.19664 | 2.80E-02 | 0.02348 | Adipose (METSIM) |
| DBT | 1 | -2.18664 | 2.88E-02 | 0.0458 | Adipose (METSIM) |
| MAPRE1 | 20 | 2.18509 | 2.89E-02 | 0.0367 | Adipose (METSIM) |
| TPH1 | 11 | -2.1808 | 2.92E-02 | 0.03101 | Peripheral blood (NTR) |
| VARS2 | 6 | 2.17878 | 2.94E-02 | 0.0446 | Whole blood (YFS) |
| ASPHD2 | 22 | 2.1771 | 2.95E-02 | 0.0375 | Whole blood (YFS) |
| BAT1 | 6 | -2.1761 | 2.95E-02 | 0.0387 | Peripheral blood (NTR) |
| FGL2 | 7 | -2.17646 | 2.95E-02 | 0.01546 | Whole blood (YFS) |
| VRK2 | 2 | 2.17629 | 2.95E-02 | 0.02771 | Whole blood (YFS) |
| GCH1 | 14 | 2.17625 | 2.95E-02 | 0.04167 | Adipose (METSIM) |
| C6orf129 | 6 | -2.17585 | 2.96E-02 | 0.04878 | Peripheral blood (NTR) |
| PIGZ | 3 | -2.17439 | 2.97E-02 | 0.04545 | Adipose (METSIM) |
| PKP4 | 2 | -2.17032 | 3.00E-02 | 0.02759 | Peripheral blood (NTR) |
| HIPK2 | 7 | -2.16973 | 3.00E-02 | 0.00862 | Whole blood (YFS) |
| MRE11A | 11 | 2.1692 | 3.01E-02 | 0.00929 | Peripheral blood (NTR) |
| GNG5 | 1 | -2.16869 | 3.01E-02 | 0.04444 | Whole blood (YFS) |
| BAIAP2L2 | 22 | 2.1675 | 3.02E-02 | 0.0117 | Whole blood (YFS) |
| AP001816.1 | 4 | -2.16287 | 3.06E-02 | 0.04898 | Adipose (METSIM) |
| SAAL1 | 11 | -2.16183 | 3.06E-02 | 0.03352 | Whole blood (YFS) |
| ULK3 | 15 | 2.1599 | 3.08E-02 | 0.0438 | Adipose (METSIM) |
| COX16 | 14 | 2.15884 | 3.09E-02 | 0.0375 | Adipose (METSIM) |
| ZMYM5 | 13 | 2.1549 | 3.12E-02 | 0.01491 | Adipose (METSIM) |
| ZMAT3 | 3 | -2.15438 | 3.12E-02 | 0.02464 | Whole blood (YFS) |
| MRE11A | 11 | 2.15374 | 3.13E-02 | 0.0102 | Adipose (METSIM) |
| ZCCHC11 | 1 | -2.14707 | 3.18E-02 | 0.00952 | Whole blood (YFS) |
| CDHR1 | 10 | -2.14625 | 3.19E-02 | 0.04478 | Adipose (METSIM) |
| MRPS35 | 12 | 2.14507 | 3.20E-02 | 0.0223 | Adipose (METSIM) |
| C10orf25 | 10 | 2.1392 | 3.24E-02 | 0.00762 | Adipose (METSIM) |
| NEK1 | 4 | -2.13977 | 3.24E-02 | 0.0387 | Peripheral blood (NTR) |
| SBNO1 | 12 | 2.13622 | 3.27E-02 | 0.04528 | Whole blood (YFS) |
| CLDND2 | 19 | 2.132237 | 3.30E-02 | 0.048 | Whole blood (YFS) |
| MSH6 | 2 | 2.13184 | 3.30E-02 | 0.02871 | Whole blood (YFS) |
| CSK | 15 | 2.131203 | 3.31E-02 | 0.0404 | Whole blood (YFS) |
| ZNF496 | 1 | 2.131 | 3.31E-02 | 0.03909 | Adipose (METSIM) |
| ASCC3 | 6 | 2.1308 | 3.31E-02 | 0.00787 | Adipose (METSIM) |
| ZMIZ2 | 7 | -2.12576 | 3.35E-02 | 0.0169 | Peripheral blood (NTR) |
| TOR1AIP1 | 1 | -2.12102 | 3.39E-02 | 0.03015 | Peripheral blood (NTR) |
| IPPK | 9 | -2.1184 | 3.41E-02 | 0.01749 | Peripheral blood (NTR) |
| GYG1 | 3 | 2.11694 | 3.43E-02 | 0.04743 | Whole blood (YFS) |
| PIGZ | 3 | -2.1151 | 3.44E-02 | 0.04669 | Whole blood (YFS) |
| NUTM2D | 10 | 2.11136 | 3.47E-02 | 0.0257 | Adipose (METSIM) |
| SYS1 | 20 | 2.1094 | 3.49E-02 | 0.04762 | Peripheral blood (NTR) |
| VAPA | 18 | -2.1089 | 3.50E-02 | 0.04878 | Whole blood (YFS) |
| CACNA2D3 | 3 | -2.10818 | 3.50E-02 | 0.03429 | Whole blood (YFS) |
| PTGDR | 14 | 2.10585 | 3.52E-02 | 0.02321 | Adipose (METSIM) |
| GPR88 | 1 | 2.10543 | 3.53E-02 | 0.03419 | Adipose (METSIM) |
| TMEM159 | 16 | 2.10177 | 3.56E-02 | 0.04633 | Whole blood (YFS) |
| A2LD1 | 13 | -2.1007 | 3.57E-02 | 0.04138 | Peripheral blood (NTR) |
| GPBP1 | 5 | 2.1004 | 3.57E-02 | 0.01657 | Whole blood (YFS) |
| BARD1 | 2 | -2.09956 | 3.58E-02 | 0.01693 | Adipose (METSIM) |
| UHRF1BP1 | 6 | 2.09971 | 3.58E-02 | 0.04494 | Peripheral blood (NTR) |
| LMO4 | 1 | -2.09656 | 3.60E-02 | 0.02791 | Whole blood (YFS) |
| INPP5E | 9 | 2.09646 | 3.60E-02 | 0.03448 | Whole blood (YFS) |
| COPG1 | 3 | 2.0955 | 3.61E-02 | 0.0002 | Adipose (METSIM) |
| TREML2 | 6 | 2.09417 | 3.62E-02 | 0.02581 | Whole blood (YFS) |
| UHRF1BP1 | 6 | 2.09227 | 3.64E-02 | 0.01451 | Whole blood (YFS) |
| COG1 | 17 | -2.09083 | 3.65E-02 | 0.02837 | Whole blood (YFS) |
| DISP2 | 15 | -2.0897 | 3.66E-02 | 0.01951 | Adipose (METSIM) |
| LYPD2 | 8 | -2.08424 | 3.71E-02 | 0.02721 | Adipose (METSIM) |
| CEP68 | 2 | 2.08254 | 3.73E-02 | 0.048 | Whole blood (YFS) |
| EIF3F | 11 | -2.08136 | 3.74E-02 | 0.04286 | Whole blood (YFS) |
| TNFRSF1B | 1 | 2.07841 | 3.77E-02 | 0.0404 | Peripheral blood (NTR) |
| KLRC3 | 12 | 2.07743 | 3.78E-02 | 0.0396 | Peripheral blood (NTR) |
| BAG5 | 14 | 2.0759 | 3.79E-02 | 0.03593 | Peripheral blood (NTR) |
| VASH1 | 14 | 2.0747 | 3.80E-02 | 0.0192 | Whole blood (YFS) |
| JUN | 1 | -2.07343 | 3.81E-02 | 0.04478 | Peripheral blood (NTR) |
| SERPINC1 | 1 | -2.07215 | 3.83E-02 | 0.0012 | Adipose (METSIM) |
| HSD17B4 | 5 | 2.0704 | 3.84E-02 | 0.00471 | Whole blood (YFS) |
| RP11-1017G21.5 | 14 | 2.06989 | 3.85E-02 | 0.03896 | Adipose (METSIM) |
| RBM44 | 2 | 2.06061 | 3.93E-02 | 0.03261 | Adipose (METSIM) |
| HERC1 | 15 | 2.0614 | 3.93E-02 | 0.03896 | Adipose (METSIM) |
| CROCCP2 | 1 | 2.05876 | 3.95E-02 | 0.04225 | Adipose (METSIM) |
| KHNYN | 14 | 2.0582 | 3.96E-02 | 0.04494 | Peripheral blood (NTR) |
| ENPP6 | 4 | 2.05806 | 3.96E-02 | 0.0443 | Peripheral blood (NTR) |
| ZNF358 | 19 | -2.057832 | 3.96E-02 | 0.0208 | Whole blood (YFS) |
| SSBP2 | 5 | -2.0572 | 3.97E-02 | 0.03625 | Whole blood (YFS) |
| SRPK1 | 6 | -2.0563 | 3.98E-02 | 0.02162 | Adipose (METSIM) |
| CTSH | 15 | -2.056 | 3.98E-02 | 0.03023 | Peripheral blood (NTR) |
| CDA | 1 | 2.05447 | 3.99E-02 | 0.02367 | Whole blood (YFS) |
| THBS1 | 15 | -2.0522 | 4.01E-02 | 0.02198 | Peripheral blood (NTR) |
| DLX6 | 7 | -2.05199 | 4.02E-02 | 0.0391 | Peripheral blood (NTR) |
| SUMO3 | 21 | -2.04606 | 4.08E-02 | 0.0404 | Whole blood (YFS) |
| NFU1 | 2 | 2.04496 | 4.09E-02 | 0.01332 | Adipose (METSIM) |
| PDIA6 | 2 | -2.03992 | 4.14E-02 | 0.03409 | Whole blood (YFS) |
| HPCAL4 | 1 | 2.03835 | 4.15E-02 | 0.00797 | Peripheral blood (NTR) |
| DEFA4 | 8 | 2.0375 | 4.16E-02 | 0.0488 | Peripheral blood (NTR) |
| TRIB3 | 20 | -2.03594 | 4.18E-02 | 0.04528 | Adipose (METSIM) |
| PCYT1A | 3 | -2.03036 | 4.23E-02 | 0.032 | Peripheral blood (NTR) |
| KAT8 | 16 | -2.0282 | 4.25E-02 | 0.0421 | Adipose (METSIM) |
| RP11-23D24.2 | 3 | 2.02456 | 4.29E-02 | 0.02251 | Adipose (METSIM) |
| WSB1 | 17 | 2.01854 | 4.35E-02 | 0.03797 | Adipose (METSIM) |
| PRPF38A | 1 | -2.01193 | 4.42E-02 | 0.00514 | Peripheral blood (NTR) |
| CCDC62 | 12 | -2.00588 | 4.49E-02 | 0.01413 | Adipose (METSIM) |
| IFIT3 | 10 | 2.00272 | 4.52E-02 | 0.03834 | Whole blood (YFS) |
| TANC2 | 17 | -1.9985 | 4.57E-02 | 0.032 | Whole blood (YFS) |
| MTSS1 | 8 | 1.98995 | 4.66E-02 | 0.0329 | Whole blood (YFS) |
| GCH1 | 14 | 1.988 | 4.68E-02 | 0.04096 | Whole blood (YFS) |
| ZNF85 | 19 | 1.98688 | 4.69E-02 | 0.00432 | Adipose (METSIM) |
| PRDX3 | 10 | -1.984 | 4.73E-02 | 0.04762 | Whole blood (YFS) |
| NOTCH4 | 6 | -1.98008 | 4.77E-02 | 0.0129 | Whole blood (YFS) |
| LEPREL1 | 3 | -1.97627 | 4.81E-02 | 0.03571 | Whole blood (YFS) |
| S100A11 | 1 | -1.97096 | 4.87E-02 | 0.01259 | Whole blood (YFS) |
| RPS8 | 1 | 1.96864 | 4.90E-02 | 0.0274 | Whole blood (YFS) |
| VASH1 | 14 | 1.96707 | 4.92E-02 | 0.02178 | Adipose (METSIM) |
| BARD1 | 2 | -1.96171 | 4.98E-02 | 0.04027 | Whole blood (YFS) |
| KLHDC7A | 1 | -1.96054 | 4.99E-02 | 0.04167 | Adipose (METSIM) |
