# Supplementary figures and images for: Integrative analysis of transcriptome-wide association study and gene expression profiling identifies candidate genes associated with stroke
Source: PeerJ. 2019 Jul 29;7:e7435. doi: 10.7717/peerj.7435 (PMC6673425; doi:10.7717/peerj.7435)

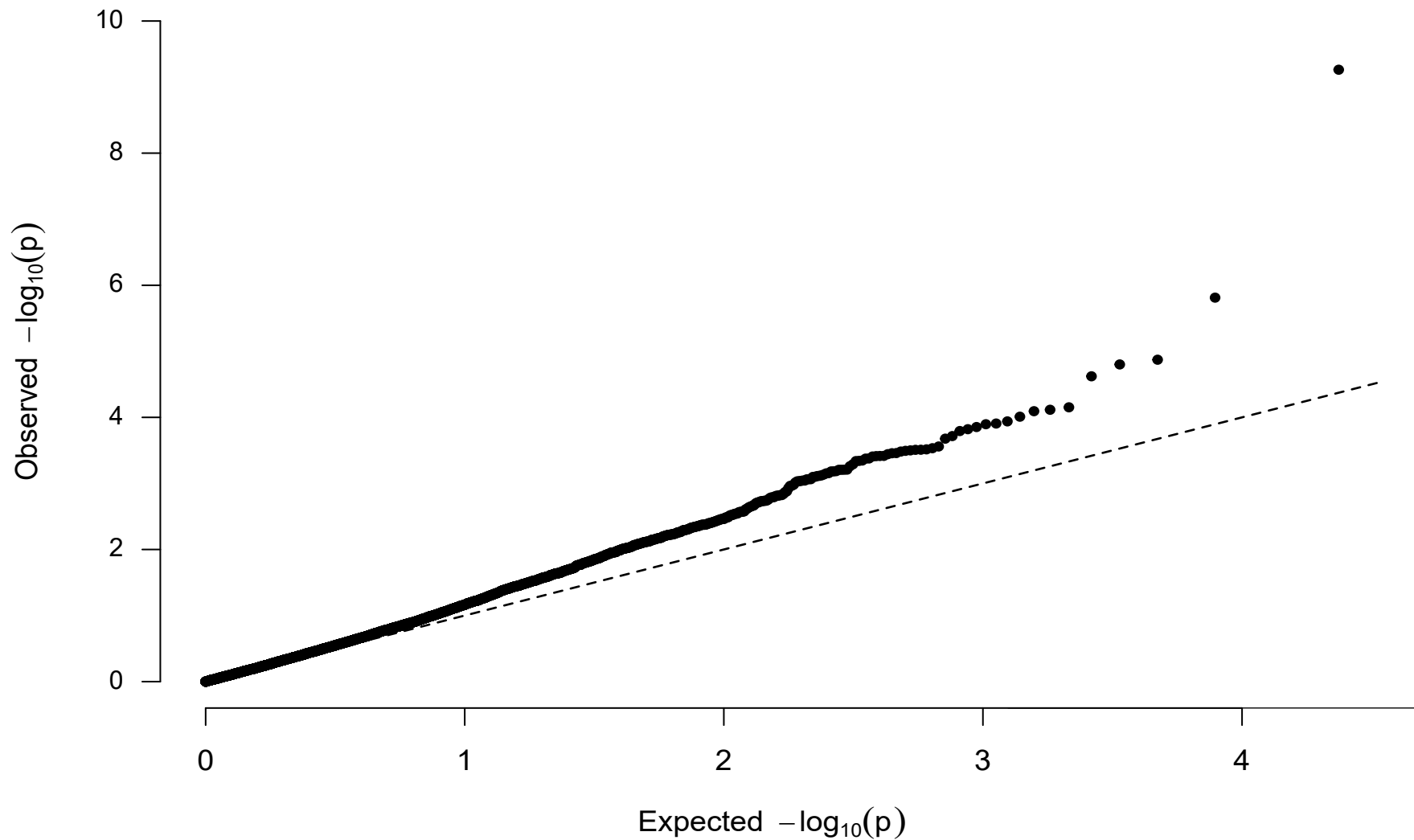

Supplement: Figure S1 [file peerj-07-7435-s002.pdf]
